# Continental-Scale Patterns Reveal Potential for Warming-Induced Shifts in Cattle Diet

**DOI:** 10.1371/journal.pone.0161511

**Published:** 2016-08-23

**Authors:** Joseph M. Craine, Jay P. Angerer, Andrew Elmore, Noah Fierer

**Affiliations:** 1 Jonah Ventures, Manhattan, KS, United States of America; 2 Texas A&M Agrilife Research, Blackland Research and Extension Center, Temple, TX, United States of America; 3 Appalachian Laboratory, University of Maryland Center for Environmental Science, Frostburg, MD, United States of America; 4 Cooperative Institute for Research in Environmental Sciences, University of Colorado, Boulder, CO, United States of America; 5 Department of Ecology and Evolutionary Biology, University of Colorado, Boulder, CO, United States of America; University of Saskatchewan, CANADA

## Abstract

In North America, it has been shown that cattle in warmer, drier grasslands have lower quality diets than those cattle grazing cooler, wetter grasslands, which suggests warming will increase nutritional stress and reduce weight gain. Yet, little is known about how the plant species that comprise cattle diets change across these gradients and whether these shifts in dietary quality coincide with shifts in dietary composition, i.e. the relative abundance of different plant species consumed by cattle. To quantify geographic patterns in dietary composition, we analyzed the dietary composition and dietary quality of unsupplemented cattle from 289 sites across the central US by sequence-based analyses of plant DNA isolated from cattle fecal samples. Overall, assuming that the percentage of reads for a species in a sample corresponds to the percentage of protein derived from the species, only 45% of the protein intake for cattle was derived from grasses. Within the Great Plains, northern cattle relied more on grasses than southern cattle, which derived a greater proportion of their protein from herbaceous and woody eudicots. Eastern cattle were also more likely to consume a unique assemblage of plant species than western cattle. High dietary protein was not strongly tied to consumption of any specific plant species, which suggests that efforts to promote individual plant species may not easily remedy protein deficiencies. A few plant species were consistently associated with lower quality diets. For example, the diets of cattle with high amounts of *Elymus* or *Hesperostipa* were more likely to have lower crude protein concentrations than diets with less of these grasses. Overall, our analyses suggest that climatic warming will increase the reliance of cattle on eudicots as protein concentrations of grasses decline. Monitoring cattle diet with this DNA-based sequencing approach can be an effective tool for quantifying cattle diet to better increase animal performance and guide mitigation strategies to changing climates.

## Introduction

There are approximately 1 billion cattle in the world with cattle populations steadily increasing over the past few decades [1]. Globally, cattle are not only important economically and societally, but they are also important ecologically as they consume a large proportion of the global net primary productivity in rangelands [2]. This consumption of natural resources is critical to the production of these animals. Over 80% of the energy and protein required by cattle to reach market weight is derived from rangeland, pasture, and other sources of roughage [3, 4].

Consumption of forage that is of low dietary quality, whether due to low protein concentrations or low digestible energy [5] can reduce weight gain and decrease reproduction. Forage quality of a mixed diet is determined by the quality of individual plant species available to cattle on the landscape, but also the mix of species consumed. Cattle are selective in their consumption of plants, which means that understanding dietary composition requires quantifying the consumption of specific species, not just their relative abundance on the landscape. For example, cattle preferentially avoid plants with low forage quality or high plant secondary metabolite concentrations [6, 7], even if those plants are relatively abundant in their grazing areas.

As climate change is likely to affect the abundances of rangeland plant species as well as their geographic distributions, climate change has the potential to disrupt livestock production by affecting the relative abundance of plant species available to cattle as well as the nutritional quality of those plant species [8]. Reduced nutritional quality with warming can result from soil warming increasing nitrogen (N) losses and reducing soil N availability, which can reduce plant N concentrations [9, 10]. Previous analyses of geographic patterns of cattle forage quality from across the portion of the conterminous US with continental climates has shown that at broad scales increasing climatic temperature decreased dietary crude protein and digestible organic matter [11]. Although warming is likely to reduce dietary quality by reducing the forage quality of individual plants, whether warming significantly alters dietary composition, i.e. the relative abundance of different plant species in the diet, is uncertain. Knowing how warming affects dietary composition could be a key to mitigating the effects of warming, or identifying vulnerabilities that could arise due to critical species’ susceptibilities to warming or other global change factors. Both changes in the abundance of species and the nutritional quality of the plant species are key components of determining the responses of herbivore diet to warming. For example, as forage quality declines, cattle may respond to the effects of warming by selecting higher quality plant species, but only if these species remain abundant on the landscape.

Determining how climate change is likely to affect dietary composition first requires techniques to quantify dietary composition. Previous research to determine cattle dietary composition has relied on direct observation of cattle, fecal microhistology, or fecal chemical analyses [12]. These techniques suffer from a suite of shortcomings including bias and low taxonomic resolution [12–15]. In contrast, metabarcoding—high-throughput sequencing of DNA to infer the assemblage of organisms contributing a sample—has a number of advantages when applied to assess dietary composition. The metabarcoding technique can be used to quantify the relative abundance of DNA of dietary items in a fecal sample and can theoretically resolve the different species that compose an animal’s diet, especially when species identification is constrained with knowledge of the local flora [16, 17]. In addition, with recent advances in DNA sequencing technologies, it is possible to use metabarcoding to quantitatively infer dietary composition from large numbers of samples relatively quickly. In the past, metabarcoding has been used to quantify the diets of a wide variety of herbivores such as coexisting large mammals, bison in Europe and North America, grasshoppers, and sheep [18–22]. For example, in North America, metabarcoding revealed that the diets of bison included a number of key species such as the N_2_-fixing eudicots like *Ceanothus* and *Lotus* that had previously been largely ignored as comprising a significant portion of their diet and that grasses comprise a greater proportion of the diet of northern bison than southern bison [23].

Despite its potential, we know of no published work using a metabarcoding approach to resolve the diets of domestic cattle in North America, and no studies that have directly quantified cattle diet across geographic gradients that could serve as analogs for future climates. Given long-standing questions on how cattle diet will shift in response to climate change and the role of individual plant species in affecting dietary quality, we assessed dietary composition and quality of 301 cattle fecal samples collected from 289 sites across the central US throughout the year. Here, we test whether there are consistent shifts in cattle diet across geographic gradients which serve as analogs for future climate. As climate gradients are likely to provide useful analogs for future climates [24–27], assessing cattle diet along climate gradients provides insight into the responses of dietary composition to changes in temperature and precipitation. Based on previous research for bison along similar geographic gradients [23], we specifically test whether the proportion of forbs and N_2_-fixing legumes in diets is greater in southern grasslands as the dietary quality of grasses declines. We also test whether there are any specific plant species that are consistently associated with higher or lower quality diets, information that could be directly relevant to understanding how to mitigate the effects of warming on cattle in the future, if not improve cattle diets independent of any changes in climate.

## Materials and Methods

Samples for analysis of cattle dietary composition were provided by the Grazingland Animal Nutrition Lab (GANLab), a commercial service and research laboratory of Texas A&M AgriLife Research [28]. Livestock producers and managers across the US collect fresh fecal samples from 5 to 10 animals within a given herd, and then mail them to GANLab fresh or frozen to be analyzed for dietary quality. Between November 14, 2014 and December 8, 2015, a total of 301 samples from 289 sites were selected from a larger stream of over 16,000 cattle fecal samples sent to GANLab. Samples were chosen for analysis haphazardly during processing of the larger flow of samples with the criteria that they did not include forage supplementation and were well-distributed geographically. For each sample selected, one subsample was frozen and stored for later DNA analysis. The rest of the sample was dried at 60°C and analyzed with near infra-red spectroscopy (NIRS) in order to estimate the crude protein ([CP]) and digestible organic matter ([DOM]) concentrations of the forage that the cattle had been consuming [28].

For DNA analysis, frozen subsamples were collectively thawed and processed together in January 2016. Genomic DNA from fecal samples was extracted using the MoBio PowerSoil htp-96 well Isolation Kit (Carlsbad, CA). Extraction blanks were included with every set of 96 samples. A portion of the chloroplast trnL intron was PCR amplified from each genomic DNA sample using the c and h trnL primers [29], but modified to include appropriate indices and adapter sequences for Illumina multiplexed sequencing. The indices used were 12-bp error-correcting indices unique to each sample [30]. Each 25 μL PCR reaction was mixed according to the Promega PCR Master Mix specifications (Madison, WI), with 2 μL of genomic DNA template. The theromocycling program used an initial step at 94°C for 2 minutes, a final extension at 72°C for 2 minutes and the following steps cycled 35 times: 2 minutes at 94°C, 1 minute at 55°C, and 30 seconds at 72°C. Amplicons from each sample were cleaned and normalized using SequalPrep Normalization Plates (Life Technologies, Carlsbad, CA) prior to being pooled together for sequencing on an Illumina MiSeq (San Diego, CA) running the 2 x 150 bp chemistry. Sequences were demulitplexed using a python script available from: https://github.com/leffj/helper-code-for-uparse/blob/master/prep_fastq_for_uparse_paired.py. Paired end reads were then merged using fastq_merge pairs [31]. Since merged reads often extended beyond the amplicon region of the sequencing construct, we used fastx_clipper to trim primer and adapter regions from both ends (https://github.com/agordon/fastx_toolkit). Sequences lacking a primer region on both ends of the merged reads were discarded.

Sequences were demultiplexed, paired end reads were merged and trimmed followed by a quality control step. Sequences were quality trimmed to have a maximum expected number of errors per read of less than 0.1 and only sequences with more than 3 identical replicates were included in downstream analyses. Trimming was accomplished with *cutadapt* http://cutadapt.readthedocs.io/en/stable/guide.html. Post merging and trimming sequences that ended up also meeting requirements for taxonomy assignment had a size range of 64–194 bps. Sequences were clustered into operational taxonomic units (OTUs) at the ≥ 97% sequence similarity level and sequence abundance counts for each OTU were determined using the usearch7 approach [32]. Here, an OTU represents a set of taxa that share similar trnL sequences grouped together for the purposes of analyses. The species that are represented by each OTU was determined by running BLASTN 2.2.30+ locally, with a representative sequence for each OTU as the query and the current National Center for Biotechnology Inventory nt nucleotide and taxonomy database as the reference. The tabular BLAST hit tables for each OTU representative were then parsed so only hits with > 97% query coverage and identity were kept.

The National Center for Biotechnology Information (NCBI) genus names associated with each hit were used to populate the OTU taxonomy assignment lists. On average, we obtained an average 19687 reads per sample after filtering and all samples had at least 2577 reads per sample. Extraction blanks averaged 1794 reads, 89% of which were from OTU 2585, which was not among our most abundant OTUs for the non-blank samples. All data were analyzed as the relative read abundance (RRA), i.e. the number of reads for an OTU relative to the total number of reads acquired per sample.

### Statistical analyses

For our purposes here, we restrict our analyses to those sites bounded by -92° and -115° longitude, which excludes 14 of the original 301 samples which were outside this geographic range.

OTU accumulation curves were generated in the R package *vegan* with the *specaccum* function using the Lomolino function to describe the curves. The percentage of graminoids, Fabaceae species, and non-Fabaceae eudicots were calculated as the sum of all OTUs identified in each group relative to the sum of all OTUs in all three groups, which excludes all unidentified OTUs. For these calculations, only the top 50 OTUs were considered, which represents on average approximately 75% of total reads. A principal components analysis (PCA) was conducted with the relative abundance of the top 50 OTUs. The PCA was conducted on the correlations of the OTUs and then the top three axes were rotated with a Varimax rotation to strengthen contrasts. A stepwise multiple regression with *P* = 0.05 was used to assess the importance of latitude, longitude, and forage quality ([CP] and [DOM]) on each of the rotated axes. Separate piecewise regression models that included the mean annual temperature and mean annual precipitation of sites showed little additional explanatory power in any regressions as mean annual temperature and latitude are highly correlated (*r* = -0.91) as well as mean annual precipitation and longitude (*r* = 0.75). As models with climate explained less variation than those based on geographic coordinates, only the results of the models that did not include climate parameters are included here for simplicity. On one axis, we used a piecewise regression model to identify non-linearities in relationships with latitude and the resultant axis scores. To assess the contribution of individual species to overall dietary quality, we engaged a stepwise regression with *P* = 0.01 that included latitude, longitude, day of year (DOY) of sampling, and the relative abundance of the top 50 OTUs. There appeared to be no effect of constraining DOY of sampling to DOY 100–250 or including all dates outside of this range. As such, samples were not constrained by DOY of sampling. All statistics except the species accumulation curves were performed in JMP 12.1.0 (SAS Institute, Cary, NC).

## Results

On average, of the most abundant OTUs in the diets of cattle sampled here, 12% of all reads for all OTUs were from species similar to species in the genus *Elymus* and 5% were from species similar to species in the genus *Bromus* (Table 1). Among the most abundant eudicot OTUs, 5% of the reads were from a *Rubus* OTU, 4% were from a *Beta* OTU and 3% were from an *Amaranthus* OTU. Overall, 25% of the reads were from the top 4 OTUs, 50% from the top 17 OTUs and 75% from the top 51 OTUs. Of the summed abundances of the top 50 OTUs, 45% of the reads were from Poaceae, 13% were from Polygonaceae, 10% were from Amaranthaceae, and 8% from Fabaceae. Examining the pattern of accumulation of OTUs, OTU abundance is predicted to asymptote at 1697 samples with half of this occurring in 10.1 samples.

**Table 1:** Abundance and multivariate patterns of top OTUs.

OTU	RRA	Representative Genus	Family	Axis 1	Axis 2	Axis 3
4045	12.1%	*Elymus*	Poaceae	0.24	0.41	-0.28
3908	4.9%	*Bromus*	Poaceae	-0.33	0.75	-0.24
51	4.3%	*Rubus*	Rosaceae	0.00	0.07	0.38
23	3.8%	*Beta*	Amaranthaceae	-0.15	-0.44	-0.31
61	2.7%	*Amaranthus*	Amaranthaceae	-0.12	-0.30	-0.30
34	2.5%	*Rumex*	Polygonaceae	-0.03	0.13	-0.04
2735	2.5%	*Festuca*	Poaceae	0.06	0.42	0.03
3598	2.5%	*Ambrosia*	Compositae	0.20	-0.03	-0.03
12	2.2%	*Polygonum*	Polygonaceae	-0.08	0.04	0.08
56	2.1%	*Eriogonum*	Polygonaceae	0.03	-0.32	-0.01
3758	2.1%	*Sporobolus*	Poaceae	-0.01	-0.17	0.38
145	1.7%	*Malva*	Malvaceae	0.05	-0.25	-0.1
2472	1.7%	*Festuca*	Poaceae	0.15	0.00	0.18
3654	1.7%	*Bromus*	Poaceae	-0.32	0.76	-0.25
91	1.6%	*Quercus*	Fagaceae	-0.09	-0.11	0.06
31	1.5%	*Persicaria*	Polygonaceae	-0.10	0.10	0.07
81	1.5%	*Oenothera*	Onagraceae	-0.05	-0.32	-0.01
170	1.4%	*Bromus*	Poaceae	0.32	0.04	0.00
27	1.3%	*Eriogonum*	Polygonaceae	-0.06	-0.25	-0.07
43	1.3%	*Lotus*	Leguminosae	0.04	0.09	0.27
3301	1.3%	*Hoffmannseggia*	Leguminosae	-0.08	-0.16	-0.07
167	1.1%	*Hesperostipa*	Poaceae	0.78	0.09	-0.05
3906	1.1%	*Aster*	Compositae	0.19	0.11	-0.07
3986	1.0%	*Phalaris*	Poaceae	0.22	0.14	0.05
16	0.9%	*Amorpha*	Leguminosae	-0.06	0.04	0.34
2805	0.9%	*Carex*	Cyperaceae	0.62	0.2	0.21
100	0.8%	*Rhus*	Anacardiaceae	-0.01	-0.14	0.14
2797	0.8%	*Carex*	Cyperaceae	0.65	0.10	-0.21
4035	0.8%	*Setaria*	Poaceae	-0.06	-0.10	-0.04
2303	0.7%	*Digitaria*	Poaceae	0.01	-0.41	-0.23
45	0.6%	*Glycyrrhiza*	Leguminosae	0.15	0.09	0.10
83	0.6%	*Prunus*	Rosaceae	-0.09	0.06	0.40
1702	0.6%	*Carex*	Cyperaceae	0.62	0.23	0.22
3209	0.6%	*Trifolium*	Leguminosae	-0.13	0.05	0.16
3678	0.6%	*Chenopodium*	Amaranthaceae	-0.10	-0.32	-0.35
3717	0.6%	*Hordeum*	Poaceae	-0.08	0.08	-0.03
7	0.5%	*Ipomoea*	Convolvulaceae	-0.04	-0.01	-0.18
40	0.5%	*Lepidium*	Brassicaceae	-0.06	-0.15	-0.03
53	0.5%	*Medicago*	Leguminosae	-0.02	0.12	-0.24
117	0.5%	*Parthenocissus*	Vitaceae	-0.09	-0.02	0.22
298	0.5%	*Glycine*	Leguminosae	0.15	-0.02	0.07
1024	0.5%	*Atriplex*	Amaranthaceae	-0.09	-0.31	-0.34
1663	0.5%	*Persicaria*	Polygonaceae	-0.10	0.03	0.04
1986	0.5%	*Toxicodendron*	Anacardiaceae	-0.04	0.01	0.44
3193	0.5%	*Setaria*	Poaceae	-0.06	-0.04	0.04
22	0.4%	*Lespedeza*	Leguminosae	-0.05	-0.04	0.13
220	0.4%	*Hesperostipa*	Poaceae	0.74	0.07	-0.05
2026	0.4%	*Dactylis*	Poaceae	-0.09	0.02	0.06
3495	0.4%	*Chloris*	Poaceae	-0.11	-0.2	0.04
3726	0.4%	*Ammophila*	Poaceae	0.03	0.04	0.18

List of top OTUs, their average relative read abundance (RRA), a representative genus for the OTU, the taxonomic family from which the representative genus resides, and the coefficients for the OTU in the eigenvectors of the top three PCA axes.

**Fig 1:**
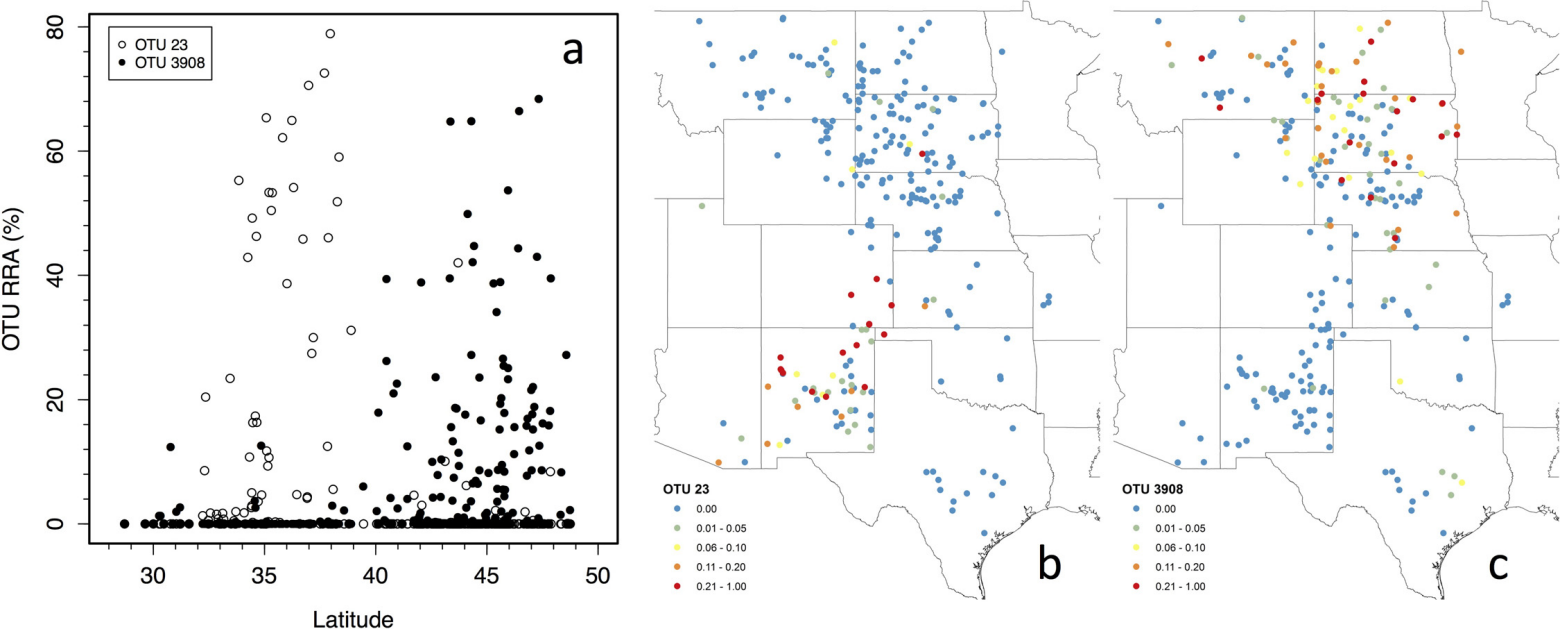
Geographic patterns of dietary composition. Relative read abundance (RRA) for (a) OTU 23 (*Beta*) and OTU 3908 (*Bromus*) in the diet of cattle as a function of latitude, as well the geographic distributions of their abundances in cattle diet (b, c).

**Fig 2:**
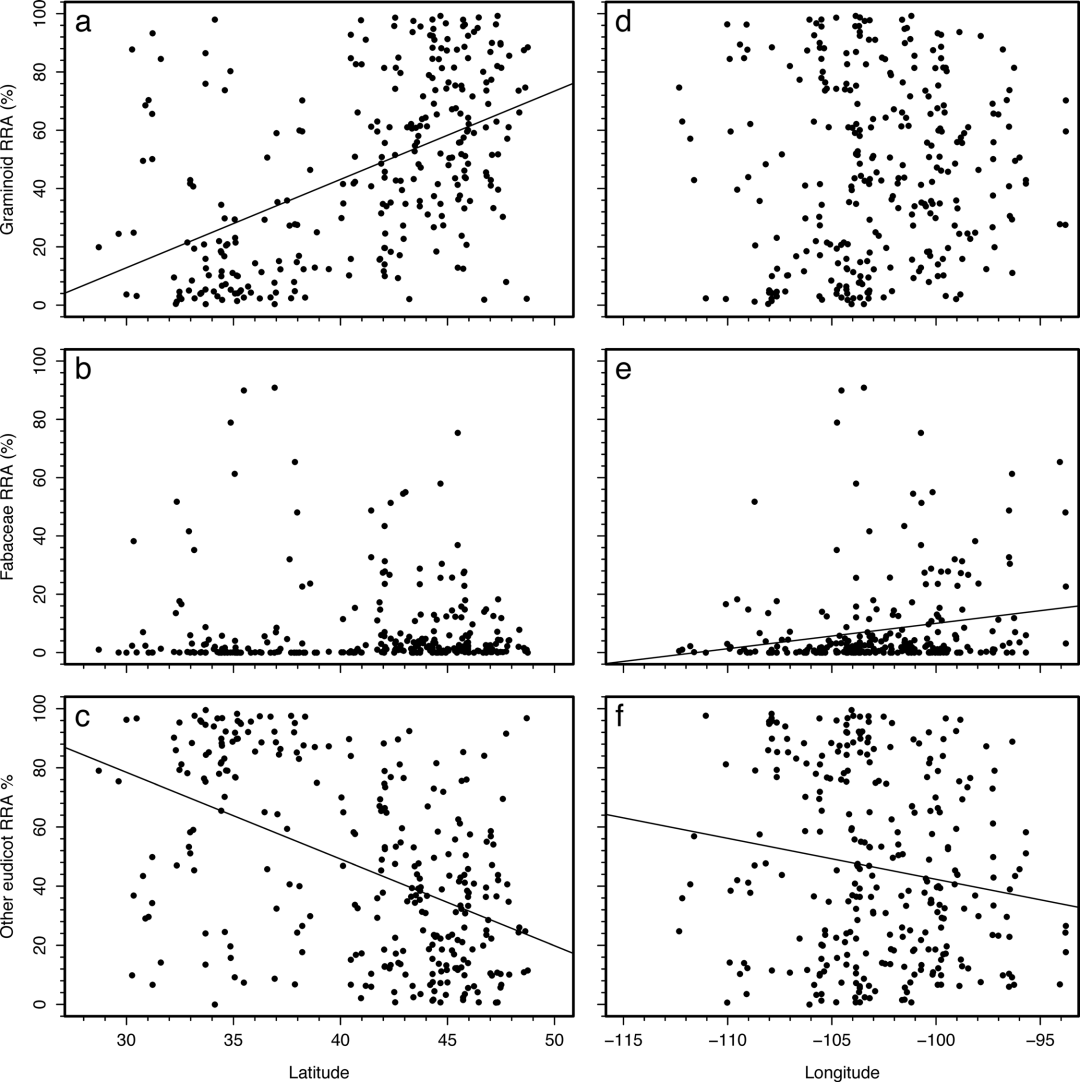
Geographic patterns of the abundance of functional groups in diets. Latitudinal and longitudinal patterns of (a,d) graminoids, (b,e) species in the Fabaceae family, and (c,f) other eudicots. Percentages expressed relative to the sum of the relative read abundances of the top 50 OTUs. Significant (*P* < 0.05) bivariate linear relationships shown. Regression results include a) r^2^ = 0.26, *P* < 0.001; c) r^2^ = 0.23, *P* < 0.001; e) r^2^ = 0.04, *P* < 0.001; and f) r^2^ = 0.03, *P* = 0.004.

The top 50 OTUs were non-randomly distributed in space and time as both latitude and day of year explained the relative abundances of OTUs (*P* < 0.001 for both). As examples of differences in species abundances along the latitudinal gradient, an OTU representing species similar to species in the genus *Beta* (or related plant taxa in the family Amaranthaceae) comprised a significant proportion of the diet of southern cattle. The consensus sequence for this OTU, i.e. OTU 23, best matched with *Beta vulgaris* at the 97% identity level over 94% of the sequence, which suggests that the species that comprise the OTU are in the Amaranthaceae family, though might not be of the genus *Beta*. In contrast, an OTU representing *Bromus* species (OTU 3908), comprised a large proportion of the diet of northern cattle (Fig 1). Overall, northern cattle consumed a greater proportion of graminoids than southern cattle. For example, cattle at 45° N had RRAs that were 43.8 ± 4.5% (s.e.) more graminoids in their diet than those at 30° N (Fig 2). Eastern cattle consumed more Fabaceae than western cattle (*P* = 0.004) with cattle at 95° W having an RRA that was 8.8 ± 3.6% (s.e.) more Fabaceae than cattle at 110° W.

**Fig 3:**
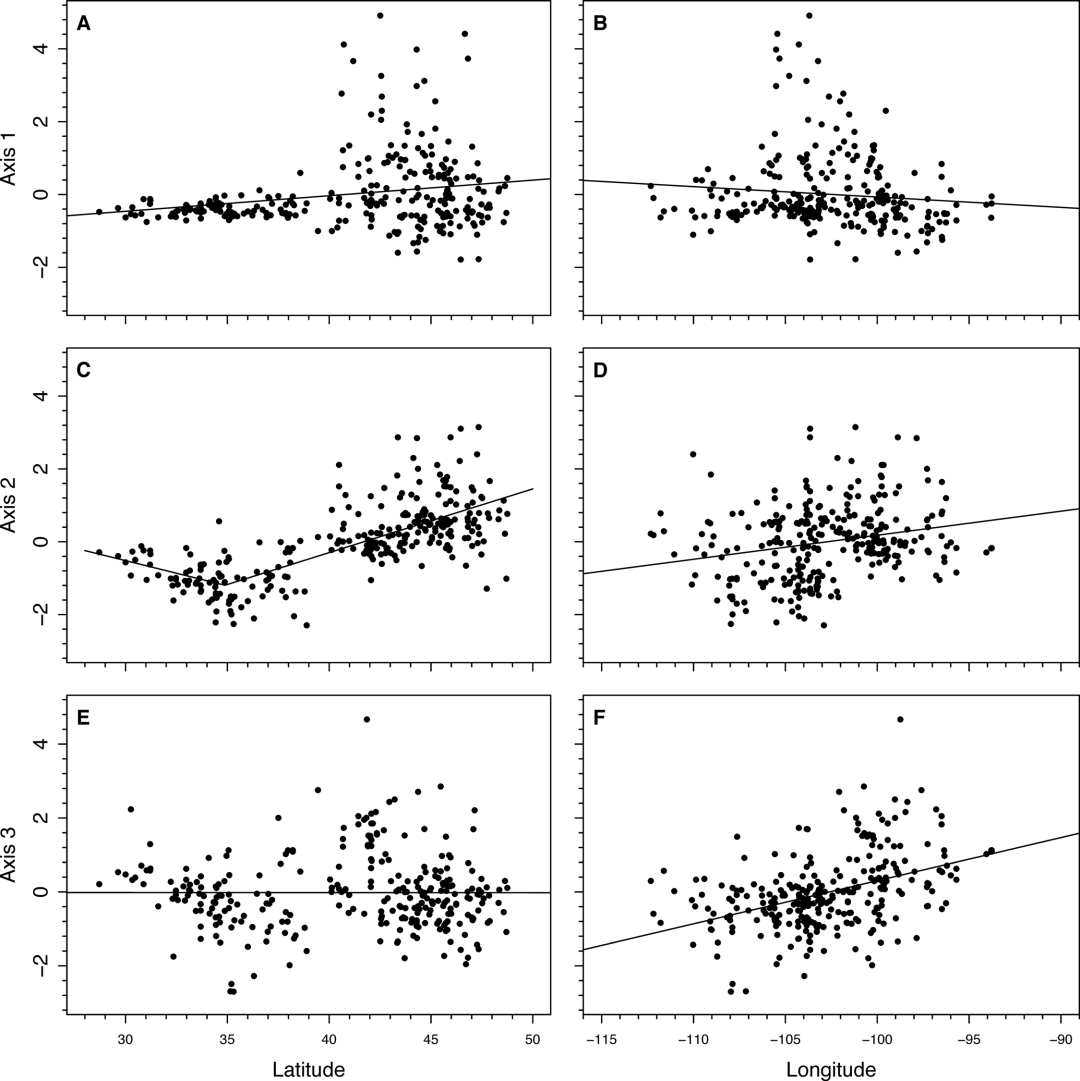
Scores of samples from the top three PCA axes shown relative to latitude and longitude. Piecewise regression were used for Axis 2 vs. latitude, but linear regression for the rest. Significant (*P* < 0.05) regression results include a) r^2^ = 0.05; *P* < 0.001; b) r^2^ = 0.48, *P* < 0.001; e) r^2^ = 0.06, *P* < 0.001; f) r^2^ = 0.17, *P* < 0.001.

Examining the multivariate relationships among the relative abundances of plants in cattle diet, the top 3 multivariate axes explained 15.9% of all variation in OTU abundance (Table 1). The first PCA axis explained 6.2% of the variation in OTU abundance (Table 1). This axis primarily differentiated cattle experiencing low-quality diets in northern sites from other cattle (Fig 3). Cattle that scored high on Axis 1 were from higher latitudes and were more likely to have lower dietary [CP] (*P* < 0.001 for both). These cattle were more likely to have consumed a greater abundance of species similar to *Hesperostipa* and *Carex* and less of those species similar to *Bromus*.

The second PCA axis explained 5.7% of the OTU abundance variation (Table 1). This axis primarily separated the diets of cattle from high-latitude eastern sites vs. low-latitude western sites (r^2^ = 0.48, *P* < 0.001; Fig 3). The diets of cattle from high-latitude eastern sites were more likely to have high abundances of species similar to *Bromus*, *Festuca*, and *Elymus* while being less likely to have high abundances of species similar to those in the genera *Beta*, *Digitaria*, and *Eriogonum*. Piecewise regression of Axis 1 scores and latitude showed that the diets of southern cattle became less “southwestern” below 34.9 ± 0.6° latitude. As an example, cattle from southern Texas (latitude < 32°) did not appear to have consumed any species similar to *Beta*, *Digitaria*, and *Eriogonum*. Instead, the most abundant representative species in their diets were from OTUs represented by species in the genera *Quercus* (14%), *Sporobolus* (6%), and *Oxalis* (4%).

Lastly, the third PCA axis differentiated sites along a longitudinal gradient (*P* < 0.001; Fig 3). Based on regression coefficients (Table 2), cattle from eastern sites were more likely to have consumed higher abundances of species similar to those in the genera *Toxicodendron*, *Prunus*, and *Sporobolus*. Cattle at the most eastern site we examined (-92° longitude) consumed 97 mg g^-1^ more of these species than cattle at the most western site (-112° longitude). Cattle from western sites were more likely to have consumed higher abundances of species similar to those in the genera *Chenopodium*, *Atriplex*, and *Beta*. Cattle at the most western site consumed 230 mg g^-1^ more of these species than cattle at the most western site.

**Table 2:** Stepwise regression results from data on dietary [CP] and [DOM] for samples.

**[CP]**	Estimate	P
Intercept	7.92 ± 1.32	< .0001
Latitude	0.13 ± 0.03	< .0001
DOY Collected	-0.019 ± 0.003	< .0001
*Elymus* (4045)	-2.94 ± 0.97	0.0026
*Hesperostipa* (167)	-28.4 ± 5.9	< .0001
**[DOM]**		
Intercept	50.5 ± 4.9	< .0001
Longitude	-0.15 ± 0.05	0.0016
DOY Collected	-0.021 ± 0.004	< .0001
*Beta* (23)	4.58 ± 1.44	0.0017
*Hesperostipa* (167)	-22.6 ± 7.0	0.0014
*Prunus* (83)	20.3 ± 6.3	0.0013

OTU ID in parentheses.

We also examined whether any OTUs consistently contributed to diets being higher or lower quality independent of latitude, longitude, and DOY collected. For [CP], [CP] was greater for high latitude sites and samples collected earlier in the year (*P* < 0.001 for both) (Table 2). Similar to the relationships signified by Axis 1, those diets with high percentages of OTUs that included *Elymus* or *Hesperostipa* were more likely to be of lower [CP]. Compared to diets with no consumption of the focal OTUs, a diet of 20% species similar to *Elymus* had a [CP] 5.9 mg g^-1^ lower (*P* = 0.003) while a diet of 20% species similar to *Hesperostipa* had a [CP] 56.7 mg g^-1^ lower (*P* < 0.001). No OTU examined significantly promoted a higher average [CP].

[DOM] also declined with both increasing longitude and DOY collected (*P* < 0.001) (Table 2). Controlling for latitude and collection date, greater amounts of species similar to *Beta* (OTU 23) and the OTU represented by *Prunus* were associated with greater [DOM]. For example, compared to cattle consuming no species in OTU 23, cattle with a diet of 20% species in OTU 23 had a diet with a [DOM] that was 9.1 mg g^-1^ greater (*P* < 0.001). In contrast, having greater amounts of *Hesperostipa* OTU was associated with lower amounts of [DOM]. For example, cattle with a diet of 20% species similar to *Hesperostipa* had a [DOM] that was 45.1 mg g^-1^ lower than cattle with no *Hesperostipa* OTU in the diet.

## Discussion

The research presented here is the first continental-scale analysis of the geographic pattern of the diet of cattle, no less any herbivore. In assessing the dietary composition of cattle across geographic gradients, our evidence suggests that as climates warm, cattle diet will become increasing more reliant on eudicots as forage quality declines. Whether this would be caused by increasing abundance of eudicots or a greater preference for eudicots is untested. The pattern of high utilization of eudicots in southern grasslands parallels other isolated reconstructions of cattle diet using other tools [33, 34]. The pattern of greater reliance on eudicots in warmer grasslands also parallels observed differences in bison at two sites within the same gradient, where the proportion of protein from grasses in the diet of bison in the southern site was half of its proportion in the northern site [23]. Assuming that this pattern is general, presumably the nitrogen concentration of grasses is lower in warmer climates causing cattle to compensate by selecting higher quality eudicots, despite the presence of secondary compounds.

Despite these patterns in relative utilization of different species, more research will be necessary to determine the relative amounts of biomass intake of different species. Because the metabarcoding approach described here targets a chloroplast gene, it likely is reconstructing the relative intake of protein from different species rather than biomass, although has matched well with biomass in the past [16]. Whether microhistology of fecal samples will provide better estimates of biomass intake of different groups of species is unknown. Microhistology is known to underrepresent high-quality species that have easily digestible cell walls [12, 14], which might be overestimating the reliance of cattle on graminoids. A recent synthesis of microhistological reconstructions of cattle diet in western US rangelands suggested that >70% of the diet of cattle was typically from graminoids [35], but whether this figure is biased by relative digestion rates of graminoids vs. herbaceous and woody eudicots is untested. Feeding trials that compare intake of different species with reconstructed dietary composition from metabarcoding and microhistology will be necessary to see the relative strengths of each approach.

Despite this general trend for greater consumption of eudicots in southern sites than northern sites, there are still northern sites where cattle are consuming high abundances of eudicots (Fig 2). For example, at one site in Montana, 65% of the protein intake was from species similar to *Amaranthus*, and 12% from *Medicago*. At another site in Montana, 69% of the protein was coming from species similar to *Amorpha* and 5% from species similar to *Rubus*. Likewise, some Texas sites registered cattle with diets that had a high proportion of grasses (Fig 2), which could be from the cattle grazing on improved pastures, but appear to be a consequence of there being a high proportion of unknown eudicots that inflated the apparent percentage of graminoids in the diet. Not only are there specific examples that run counter the general trend, the shifts in diet in the southern Great Plains reinforces that shifts in diet are not unimodal along these latitudinal gradients. At a latitude of approximately 35°N, as one progresses southward, the dietary percentage of species such as *Digitaria* and *Chenopodium* stop increasing and other species such as *Quercus* and *Sporobolus* increase, likely reflecting the shift in vegetation from southern grasslands to ecoregions such as live oak savannas of Texas.

Independent of its relevance for climate change, monitoring cattle diet can serve as a benefit to producers in a number of ways. For example, grasslands where cattle consume greater amounts of *Hesperostipa* are associated with low dietary quality. Species like *Hesperostipa* typically can have low foliar N concentrations and often are not considered high-quality forage [36, 37]. It is uncertain at this point whether consumption of these species in and of themselves reduces dietary quality or whether their presence in the diet is indicative of sites with lower forage quality. Regardless, knowledge of which plant species cattle are consuming at different times of year can either set the stage for improvements or managing species that are critical to diet at different times of year. In addition, the technique has the potential to identify toxic plants in cattle diet, which may help prevent acute toxicity, if not identify the cause of toxicity syndromes if consumption of toxic plants is high enough. For example, as much as 28% of the reads of a sample were from OTU 45, which matches to toxic species such those in the genera *Astragalus* (though the European non-toxic *Astragalus cicer* is grown as forage in the northern Great Plains), and *Oxytropis*, which can cause neurologic disorder when consumed in sufficient quantity. The technique also holds promise for illuminating how cattle diet shifts with other global change factors such as drought, as well as with grazing intensity or between breeds of cattle when working to optimize utilization of plants on the landscape.

Planning for the future, the metabarcoding technique shows obvious advantages over other techniques for diet reconstruction that will aid in further understanding how herbivore diet responds to environmental change and fashioning mitigation strategies. Compared with microhistology, metabarcoding requires less effort and specialized training as well as greater taxonomic resolution. There are limitations to the metabarcoding technique, but some of these can be strengths under some circumstances. Although dietary composition from metabarcoding should be influenced by the protein concentrations of individual species consumed, this limitation is also a strength in some cases. Scaling RRA with trnL to biomass intake can be done if relative protein concentrations of different species are known, yet reconstructing relative protein intake might be more desirable than biomass intake for the purposes of nutrition since most cattle are protein-, not energy-, limited [11].

Still, using the trnL approach does not resolve all species, no less all genera. For example, the most abundant OTU, OTU 4045, could have represented species from over 40 genera of grasses, such as *Elymus*, *Agropyron*, *Triticum*, *Leymus*, *Festuca*, or *Poa*. Pairing trnL with other sequences will be necessary in the future to provide greater taxonomic resolution [16]. In addition, there still is a need for more collections, as indicated by the uncertainty associated with OTU 23. The best match for OTU 23 was with *Beta vulgaris*, but the differences between trnL for *Beta vulgaris* and the consensus sequence for OTU 23 suggest that it was not actually *Beta vulgaris*. The plant species with next highest closest matches for OTU 23 were also in the Amaranthaceae, but are not present in our study region. These included species such as *Arthrocnemum macrostachyum* (98% match over 92% of the sequence), which is native to North Africa, and *Dysphania melanocarpa* (98% match over 91% of the sequence), which is native to Australia. Multiple species in the Amaranthaceae genera of *Amaranthus*, *Atriplex*, *Chenopodium*, *Gomphrena*, and *Tidestromia* have been sequenced for trnL, but not all species of these genera, or other genera in Amaranthaceae found in the region have been sequenced. So, at this point, it appears that cattle in New Mexico and Colorado consume species in the Amaranthaceae, yet further collection and sequencing of Amaranthaceae species will be necessary to ultimately identify this key component of the diet of cattle in the region.

## Conclusions

In all, given the geographic patterns in diet, it appears that as climates warm, cattle will respond in part by increasing their relative consumption of protein from high-protein eudicots to compensate for declines in protein concentrations in grasses. As protein concentrations appear to have already been declining in grasslands for decades due to increasing atmospheric CO_2_ concentrations [38], warming and elevated CO_2_ concentrations are likely reinforcing nutritional stress in cattle, though the degree to which cattle nutritional stress may have increased is currently unquantified. Monitoring the diet of cattle likely will be an important component in responding to climate-induced nutritional stresses as increases in forb component of diets might indicate elevated nutritional stress. Mitigating nutritional stress will likely require altered management practices such as promoting high-protein plant species, altered use of fire [39], or increasing protein concentrations of grasses, possibly through fertilization. With increases in eudicot percentages in diets, more attention will need to be paid to the role of secondary compounds in dietary selection and nutrition [6]. Given the patterns observed here, some management practices, such as broad-scale application of herbicides to reduce eudicot abundance, might be deleterious nutritionally and an improper response to reduced nutritional quality of forage.
